# fMRI-based neurofeedback strategies and the way forward to treating phasic psychiatric symptoms

**DOI:** 10.3389/fnins.2023.1275229

**Published:** 2023-12-06

**Authors:** Candela Donantueno, Pierre Yger, François Cabestaing, Renaud Jardri

**Affiliations:** ^1^University of Lille, INSERM U-1172, CHU Lille, Lille Neuroscience & Cognition Center, Plasticity & SubjectivitY Team, Fontan Hospital, Lille, France; ^2^University of Lille, CNRS, Centrale Lille, UMR 9189 CRIStAL, Lille, France

**Keywords:** hallucinations, neurofeedback, fMRI, multi-voxel pattern analysis, coping, neuromodulation

## Abstract

Auditory verbal hallucinations (AVH) are the perfect illustration of phasic symptoms in psychiatric disorders. For some patients and in some situations, AVH cannot be relieved by standard therapeutic approaches. More advanced treatments are needed, among which neurofeedback, and more specifically fMRI-based neurofeedback, has been considered. This paper discusses the different possibilities to approach neurofeedback in the specific context of phasic symptoms, by highlighting the strengths and weaknesses of the available neurofeedback options. It concludes with the added value of the recently introduced information-based neurofeedback. Although requiring an online fMRI signal classifier, which can be quite complex to implement, this neurofeedback strategy opens a door toward an alternative treatment option for complex phasic symptomatology.

## 1 Introduction

Encountering groups of individuals for whom conventional psychiatric and psychological treatment paths have been insufficiently successful, such as pharmacotherapy (Elkis, 2007) or Cognitive Behavioral Therapy (CBT) (Bighelli et al., 2018), is unfortunately a common issue. Current medications and psychological therapies are not effective for everyone, and also not all the time. And this represents a rather large problem from our perspective. Therefore, we see an active quest for new treatments that help those who have not been already helped by the more traditional treatment routes and approaches, like neurofeedback (NF), which are becoming increasingly popular as potential alternative treatments (Fovet et al., 2016).

NF is a neuromodulation method that allows participants to be more in control of the parts of their own brain activity that are related to a specific function or disorder. The goal is for the subject to become aware of the changing brain’s activation thanks to easily understandable feedback. Consequently, participants are trained and therefore expected to be able to monitor their progress in the task of modulating their own brain activity, usually by either up or downregulating this activity (Marzbani et al., 2016). Moreover, a trained participant can continue with this self-regulation even when the feedback is not provided anymore (Humpston et al., 2020). So far, the most researched routes of administration, speaking from a technological point of view, have been electroencephalogram – (EEG) and fMRI-based NF (Arns et al., 2017).

NF strategies have so far been explored mostly in the methodological context of EEG, where the temporal resolution is superior to the fMRI-based NF technique, but its spatial resolution is significantly inferior. The increased spatial resolution of fMRI-based NF allows researchers to target particular regions and networks in the brain, by delimiting these with structural or functional brain localizers (Thibault et al., 2018; Humpston et al., 2020). This represents a strong advantage when compared to EEG methods for NF therapies that are meant to treat disorders involving deeper brain areas, such as auditory verbal hallucinations (AVH), a common symptom of schizophrenia.

When discussing AVH in the present paper, we will be referring to “clinical voices,” as already described in the literature by Toh et al. (2022). Hearing these voices refers to individuals hearing speech (often with non-verbal sounds). This type of experience usually starts occurring during adolescence or early adulthood. The frequency of the voices seems to be higher than in “non-clinical voices.” There seems to be less perceived control over the voices, and they also seem to be perceived as threatening more often. The levels of emotional distress are also more elevated, and this has an impact on daily functioning (Toh et al., 2022).

**Figure 1:**
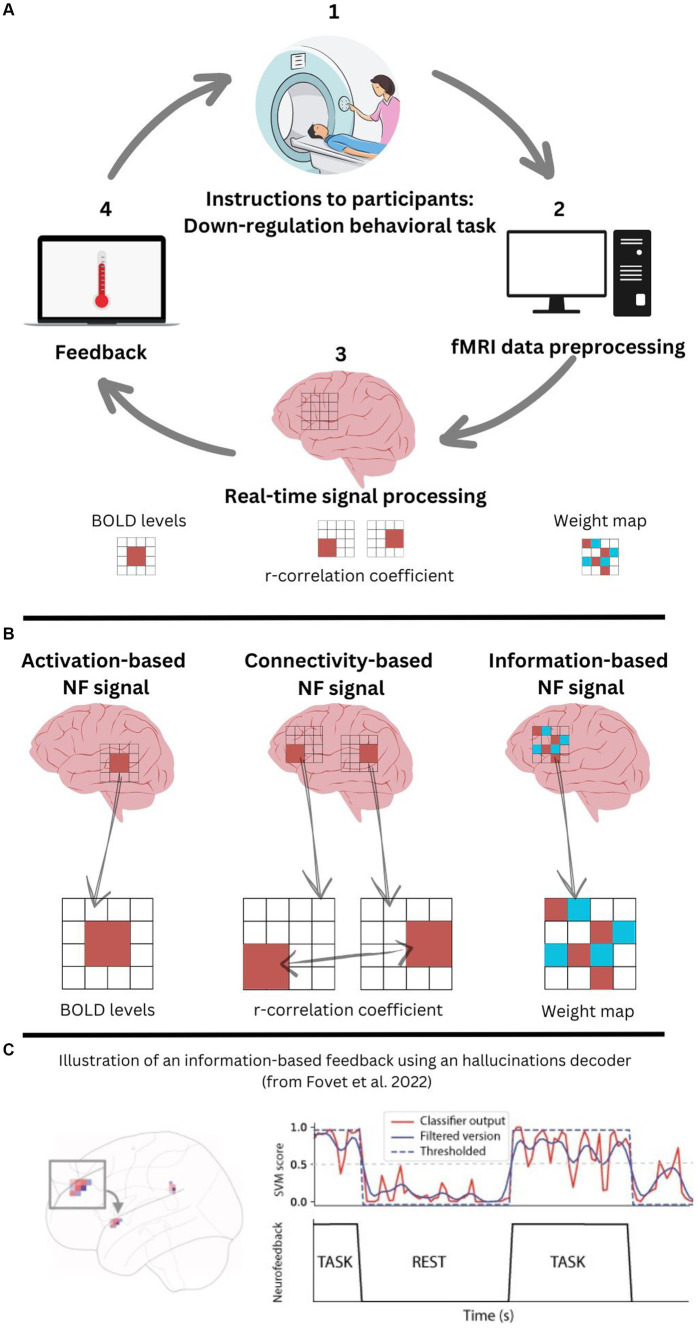
**(A)** fMRI-based neurofeedback loop; **(B)** Comparison of the three fMRI neurofeedback methods; **(C)** Information-based approach for fMRI-based neurofeedback to treat auditory verbal hallucinations.

In order to conduct an fMRI-based NF experiment, a neural signal – traditionally a blood oxygen level-dependent (BOLD) signal – from a region of interest (ROI) is necessary. This is then processed in real-time and consequently fed back to the participant, usually through visual feedback (e.g., participants can be asked to “bring down a rocket to Earth,” or to “lower the temperature of a thermometer,” *cf.* Figure 1A). Other sensory modalities can be employed as well to provide the feedback. The participant is then encouraged to engage in a coping strategy that was pre-convened, with the goal to self-regulate their brain activity (Fovet et al., 2016; Orlov et al., 2018).

The first result from research studies showing that fMRI-based NF is an effective technique to treat complex symptoms was conducted on chronic pain (deCharms et al., 2005). Pain symptomatology is caused not only by an objective sensory input but also largely by the subjective perception from the patient’s perspective and experience of it. This line of research opened a door for the investigation of NF methods for the more subjective symptoms in the field of psychiatry.

fMRI-based NF has also been employed as an alternative or add-on therapy for different types of symptoms across psychiatric disorders. A recent meta-analysis summarizes the various psychiatric disorders for which fMRI-based NF could be helpful (Pindi et al., 2022). Depression remains the main researched psychiatric disorder to date in the fMRI-based NF interventions (Linden et al., 2012; Mehler et al., 2018). Crucially, depressive mood is classified as a “tonic symptom” since it is present and active most of the time and is also part of the baseline of the person’s mental functioning. Opposite to tonic symptoms are “phasic symptoms,” which are appearing intermittently and are usually triggered by environmental cues, such as hallucinations in disorders like schizophrenia (Hartman and Burgess, 1993). fMRI-based NF can be used to treat different types of symptoms, and both tonic and phasic symptoms can be the target of such an intervention. Among the disorders explored by Pindi et al. (2022), schizophrenia is also presented as a disorder for which this type of fMRI-based NF could be suitable.

In about three in four patients, schizophrenia is characterized by patients experiencing AVH (Thomas et al., 2007). In this sub-group, it is estimated that approximately one in four of them do not respond to traditional antipsychotic medication (Shergill et al., 1998). Other different potential solutions have been proposed for this rather large group of patients with treatment-resistant AVH. Examples of these are Non-Invasive Brain Stimulation (NIBS) techniques, such as repetitive Transcranial Magnetic Stimulation (rTMS) or transcranial Direct Current Stimulation (tDCS), which demonstrated small-to-moderate effects in reducing hallucinations severity (Slotema et al., 2014; Mondino et al., 2018).

These alternative NIBSs are further in their development than fMRI-based NF, however, they are not instrumental to the goal of treatment of AVHs because, with rTMS or tDCS, it stays difficult to target deeper structures in a precise way. Furthermore, NF methods in general offer the intrinsic involvement of the patient in the treatment procedure by having them engage in a coping strategy while being in the scanner, and this could be considered of added value to this novel strategy to relieve AVH because it could result in a natural feeling of empowerment from the patient on their own recovery.

## 2 What are the different fMRI-based NF strategies to treat AVH?

The signal processing of fMRI-based NF treatment for AVH can be approached from different methodological strategies namely, activation-based, connectivity-based, and information-based (*cf.* Figure 1B). Table 1 aims to synthesize in a unique way what these different strategies can add from the different perspectives of patients (P) and researchers/clinicians (R/C). It is our presumption that most likely, nowadays, clinicians and researchers would prefer activation-and connectivity-based methods, given the fact that there is significantly more evidence proving these two methods effective for some groups, in some circumstances. We also presume that patients would prefer the information-based method, presumably because of the sense of agency they could have in their own recovery because of this method.

**Table 1:** Strengths and weaknesses of each neurofeedback strategy (“P”: patient, “R/C”: researcher/clinician); “2b*” corresponds to individual cohort studies/low-quality randomized control studies.

	Activation-based	Connectivity-based	Information-based
Strengths	(P, R/C) Non-invasive techniques (P, R/C) The active role of the patients (empowerment) (P, R/C) Enhances motivation and success rates		
	(P, R/C) Trait-markers, no need for in-scanner hallucinations		(P, R/C) State-markers, works on actual AVH occurrence (P, R/C) Individualized treatment options
	(P) Easy to implement (few regions targeted)	(R/C) AVH-related impairments at the network level (P) The down-regulation is more specifically focused on the AVH network symptoms	(P, R/C) Captures pattern-related activations of an active AVH episode happening (P) There is down-regulation training on the AVH event itself (R/C) A better understanding of patient-specific AVH and its nuances
Weaknesses	(P) Standardized instructions, given the hemodynamic response: patients must focus the entire session (P, R/C) NF needs to happen within an MRI scanner (P) Potential claustrophobic symptoms (R/C) More expensive and less accessible than EEG methods; a more advanced understanding of functional neuroanatomy required		
	(P) Trait-markers, the down-regulation strategy might not work on actual AVH		(P, R/C) State-markers: need for in-scanner hallucinations (P) Patients in likely distress (P) Only for those with persistent hallucinations (R/C) Difficult to find suitable candidates
	(P, R/C) Oversimplifies AVH abnormalities at the network level (R/C) AVH neuroanatomical knowledge required	(P) Does not target specific symptoms, reduced benefit (R/C) Knowledge of AVH at the network level of the AVH symptom required	(P, R/C) In the early phases of development (P) Treatment not readily available (R/C) Plenty remains unknown (R/C) Computational and algorithmic skills required
Level of evidence (Saragossi, n.d.)	2b*	2b*	No evidence yet

Activation-based NF stays the simplest approach, where the spatially averaged BOLD signal level of a region of interest is directly fed back to the participant. The goal is to increase or decrease activity in these targeted brain regions according to what we know of their role in the pathophysiology of the underlying disorder. Activation-based NF requires the neuroanatomical identification of the brain area(s) that are representative of where the AVH are originating from Orlov et al. (2018) and Okano et al. (2020). Therefore, a “localizer scan” is necessary for activation-based NF methods, and it is meant to create a functional mask, which is used to derive the fMRI-based NF signal. To create this mask, univariate fMRI analysis techniques are used when computing the signal changes in the areas that become activated by the functional task, previously localized during the first localizer scan.

Usually, activation-based NF training protocols for patients consist of a block design (Orlov et al., 2018; Okano et al., 2020), where these alternate between “rest blocks” (no regulation takes place) and “down-regulation blocks” (an active attempt to regulate). During the down-regulation blocks, participants are presented with the feedback information where they can interact with the training. In the case of the study conducted by Orlov et al. (2018), a space rocket was shown to the participants as feedback during the training blocks, and they were instructed to “bring the rocket down to Earth.” However, other types of feedback can be implemented, even in other sensory modalities such as auditory or haptic feedback (Fleury et al., 2020). The strategy employed by the participants to succeed in the task can be pre-convened or it can be left up to the participant to decide on the go what they prefer to do, and report on that to the researchers afterward. Participants need to be alerted of the inherent hemodynamic response-related delay in the feedback of about six seconds, which is due to the BOLD response, which in turn results in the need for the participants to strictly follow standardized instructions.

Three to five sessions are usually necessary for a full round of activation-based fMRI-based NF protocol, which was also the case in the study carried out by Orlov et al. (2018). The first session is necessary for the localizer scan. The following sessions are meant for the true NF training, and the final session can opt to become a “transfer run,” where the feedback signal is not presented, and participants are asked to do the task anyway, without having input from the (visual) feedback. The aim of a transfer run is to simulate real-life conditions and to test its generalizability where neural feedback is not available.

Connectivity-based NF is the next step in complexity after an activation-based approach. A correlation coefficient is calculated between different ROIs or different networks that are known to be involved in the disorder. The goal here is now to modulate the functional connectivity within or between brain networks. A functional localizer scan is also conducted in this type of NF, except that in this case, the localization is conducted on the relevant multiple networks or regions of interest, instead of just on one region (Zweerings et al., 2019; Bauer et al., 2020). In this type of NF, the structure of the training sessions can remain roughly the same, where there is an alternation of regulatory and non-regulatory blocks (i.e., rest blocks).

Information-based NF is a pioneering strategy for NF where the focus is on regulating patterns of activation in the relevant brain areas (see Fovet et al., 2022 for an illustration of functional patterns associated with AVH occurrences). This type of NF relies on “state markers,” in contrast to the former two methods described, which rely on “trait markers.” Because information-based NF focuses on the phasic or transient nature of the symptom, participants in this type of NF training need to have active AVH episodes, including during the training itself. For activation-and connectivity-based NF, this is not required (Fovet et al., 2016). The goal of the information-based strategy is to train self-regulation of brain areas that become reactivated during the occurrence of the AVH symptom itself. The neural signal used in this type of NF is the weight map predicting AVH states’ s as seen in Figure 1C.

Another important difference between the formerly described strategies and information-based NF is that it requires the use of an online decoder that can detect in real-time the occurrence of AVH. This is related to the fact that it relies upon state markers, which require participants to be actively hallucinating while in the fMRI scanner and receiving direct feedback on their brain pattern activations, with minimal delays. Researchers from our team (Fovet et al., 2022) have developed a machine-learning classifier using linear Support Vector Machine (lSVM), able to classify in a multivariate fashion the functional patterns specific to the AVH state. This classifier has been benchmarked against verbal imagery. It should be noted, however, that further data needs to be produced for this classifier to be able to capture a wider phenomenology of voice-hearing experiences.

In order to better detect active hallucinations and to enable a prompt delivery of the feedback, inner periods of hallucinations were defined and labeled for classification: from ignition to extinction (Lefebvre et al., 2016). “OFF” usually represents the periods without hallucination and “ON” is the periods with hallucinations. In this way, the algorithm was trained on large datasets to later classify and predict when someone is hallucinating or not, and/or in which part of the hallucinatory episode they find themselves. A unique fact about the algorithm previously developed is that it was built and trained with a much more favorable count of labels (i.e., hallucinatory episodes), instead of the feasible count of subjects with AVH that could take part in the data acquisition (since a given subject can hallucinate several times during a scanning session).

## 3 Discussion

Information-based NF, just as the earlier developed NF strategies, is a non-invasive neuromodulation technique that has the potential to reduce AVH in patients who do not respond as expected to antipsychotic medications, by giving them an active role in their recovery. Table 1 represents the strengths and weaknesses of the three NF methods previously discussed (i.e., activation, connectivity, and information-based), from the perspectives of the different stakeholders: the patients and the researchers/clinicians. We see in Table 1 that their interests do not always coincide when it comes to choosing a method. There seems to be a clear discrepancy, which we think could be helped with the running of a Randomized Control Trial (RCT) to test information-based methods, and in this way, bring these three stakeholders together.

The information-based NF approach has certain advantages over activation-and connectivity-based approaches to NF, which makes a potential leap forward in treating AVH. These include the fact that there are nuances to AVH features that information-based approaches can address, and the former two methods cannot. Secondly, the down-regulation task in the information-based NF approach is entirely focused on the moment the hallucinatory event is taking place (i.e., information-based NF relies upon state-markers), therefore, addressing the real AVH experience and phenomenology. Lastly, it is important to highlight the fact that the subjectivity of AVH can stand in a central role in information-based approaches that is not possible with the other two methods.

However, there are certain characteristics of an information-based approach to NF for AVH that complicate matters in a significant way. This method relies upon state-markers, requiring that the patients hallucinate very often, and more specifically, multiple times while in the scanner, drastically reducing the number of patients that can benefit from it. The classifier allows for an easier generalization in non-expert centers, regardless of whether the characteristics of the patients that can be included remain challenging.

It should not go unmentioned by the fact that there is another contemporary approach in development to information-based NF, namely “process-based NF.” This strategy represents a step forward in refining NF strategies to be able to treat more complex symptomatology (Lubianiker et al., 2019), even if it appears mainly applicable for tonic symptoms (e.g., depression), while we propose that the treatment of hallucinations (i.e., a phasic symptom) requires a different type of approach which can also account for changes at a smaller time scale.

As explained in Table 1 in the section “Levels of Evidence” (Saragossi, n.d.), there is an urgent need for preliminary data in support of the information-based approach. It has become increasingly important to develop innovations in neurotechnology that are both personalized and can target specific brain areas or networks. fMRI plays and will continue to play a critical role in both mentioned themes that require innovation.

Information-based NF is currently the only potential approach to an NF treatment that can target phasic symptoms, such as hallucinations. It simultaneously empowers patients by offering them a tool to better control their symptoms and become an agent in their own recovery. We believe that this is very much in line with the mission that the “NeurotechEU European University” is on, to couple neurotechnological advances with augmenting patients’ mental ability to respond more appropriately to the challenges they face due to their health situations.

However, before being able to use this technique, relatively good detection and classification performance levels in the fMRI scanner are required for a successful information-based NF treatment. And this is a key research challenge we are currently facing, given the well-known often comorbid cognitive symptomatology in patients from this target group.

## Author contributions

CD: Writing – original draft, Writing – review & editing. PY: Writing – review & editing, Writing – original draft. FC: Writing – original draft, Writing – review & editing. RJ: Writing – original draft, Writing – review & editing.
